# CYP1B1-AS1 Is a Novel Biomarker in Glioblastoma by Comprehensive Analysis

**DOI:** 10.1155/2021/8565943

**Published:** 2021-12-29

**Authors:** Tao Ye, Lan-lan Li, Xue-mei Peng, Qin Li

**Affiliations:** ^1^Department of Clinical Laboratory, Chongqing General Hospital, University of Chinese Academy of Sciences, Liangjiang New Area, Chongqing, China; ^2^Department of Neurology, Chongqing General Hospital, University of Chinese Academy of Sciences, Liangjiang New Area, Chongqing, China

## Abstract

**Objective:**

Growing evidence shows that enhancer RNAs (eRNAs) are pivotal for tumor progression. In this research, our team aimed to identify the survival-related eRNAs and further explore their potential function in glioblastoma (GBM).

**Methods:**

RNA-sequencing data in 31 tumor types were acquired from TCGA datasets. The survival-related eRNAs were identified by the use of Kaplan-Meier survival analyses and Spearman's correlation analyses. KEGG pathway enrichment analysis was completed to investigate the underlying signal paths of the critical eRNA. Pancancer assays were applied to explore the association between CYP1B1-AS1 and CYP1B1.

**Results:**

We identified 74 survival-related eRNAs and focused on CYP1B1-AS1 which displayed the greatest cor value. CYP1B1 was identified as a regulatory target of CYP1B1-AS1. KEGG analyses suggested that CYP1B1-AS1 might play an essential role through CK-CKR mutual effect, complement and coagulation cascades, TNF signal path, and JAK-STAT signal path. The pancancer verification outcomes revealed that CYP1B1-AS1 was related to survival in 4 cancers, i.e., LIHC, KIRP, KICH, and KIRC. Association was discovered between CYP1B1-AS1 and the targeted gene, CYP1B1, in 29 cancer types.

**Conclusion:**

The outcomes herein provided the first evidence that overexpression of CYP1B1-AS1 might be a potential molecular biomarker for predicting the prognosis of patients with GBM.

## 1. Introduction

Glioblastoma (GBM) is the most common and malignant tumor of the human central nervous system, accounting for 81% of brain tumors [1]. It is extremely aggressive, and the disease develops very fast [2]. Despite the fact that early diagnostic and therapeutic approaches of GBM have developed remarkably recently, the mean survival posterior to diagnoses is 12-15 months, with less than 5% sufferers living >5 years, primarily owing to the commonly seen late period illness with lymphatic or distant metastatic activities [3, 4]. The absence of valid treatment is mainly due to the insufficient knowledge of the molecule-level etiopathogenesis of GBM. Hence, our team wishes to determine new markers tightly associated with the carcinogenesis of GBM which can help clinical doctors when it comes to the early risk evaluation, personalized therapies, and forecast of the survival of GBM.

Thanks to the development of the latest sequencing techniques, ncRNAs have remarkably aroused our interest in the academic world owing to the fact that they are capable of regulating genetic expression [5]. eRNA pertains to ncRNA transcribed from the enhancer and serves as a specific type of lncRNA generated by transcript of enhancer elements [6]. Researches have revealed that they are vital for the mediation of targeted genetic stimulation and transcript [7, 8]. Numerous eRNAs are recognized in mankind cells, masses of which are discovered to be capable of mediating the stimulation of targeted genes [9, 10]. Some lncRNAs have been reported to participate in the proliferative ability, metastatic events, and drug resistance of a wide range of cancers [11, 12]. Although eRNA is pivotal for the transcriptional control of genes, the underlying roles of eRNA in GBM are still elusive.

In the present research, our team identified 74 survival-related eRNAs in GBM. These eRNAs may provide a new clue for the research of the potential mechanisms involved in GBM progression. Then, our attention focused on CYP1B1-AS1 whose function was rarely reported in tumors. Our findings provided evidences that CYP1B1-AS1 may be utilized as a novel treatment target and prognostic biomarker for GBM sufferers.

## 2. Materials and Methods

### 2.1. Original Data Collection and Processing

TCGA databases had collected and studied masses of clinic and molecule-level data of more than 10,000 cancer sufferers covering 33 diverse cancer types. Transcriptomic RNA-sequencing information of 33 tumors was abstracted from TCGA database. These 33 tumor types involved the following: ACC, BLCA, BRCA, COAD, DLBC, ESCA, GBM, HNSC, KICH, KIRC, KIRP, LAML, LGG, LIHC, LUAD, LUSC, OV, PAAD, PRAD, READ, SKCM, STAD, TGCT, THCA, THYM, UCEC, and UCS.

### 2.2. Identification of Predictive eRNAs in GBM by Comprehensive Data Analysis

The mankind GTF files were utilized to convert eRNA transcription IDs into genetic symbols, and the expression profiling of the eRNAs was abstracted from the RNA expression profiling of pulmonary glandular carcinoma. Subsequently, our team integrated the eRNA expression matrices with the GBM survival information via the limma R software package. The survival-related eRNAs were selected via K-M approach, and we took the FDR modified *p* < 0.05 as normal cutoff values; our team chose eRNAs meeting such criteria as survival-related eRNAs. The sufferers were separated into the expression_low_ group and the expression_high_ group as per the mean expression of every eRNA. After that, Spearman's correlation analyses were finished to acquire promising critical eRNAs associated with survival and related to targeted genes affecting GBM; cor > 0.4 and *p* < 0.001 were deemed as significant on statistics.

### 2.3. Functional and Pathway Enrichment Assay

DAVID 6.8 Bioinformatics Resources was used for Gene Ontology (GO) and KEGG pathway annotations [13]. We ran a GO enrichment analysis for targeting genes of CYP1B1-AS1 for the 3 GO domains: MF, BP, and CC. In addition, bubble charts were plotted by using the ggplot2 package of R software [14]. *p* < 0.05 was utilized as the standard.

### 2.4. Validation in Pancancer

Firstly, the expressing data of CYP1B1-AS1 and the targeted gene CYP1B1 in pancancer were acquired via the R limma package, and the expressing matrix was integrated with the survival information of pancancer. The specimens were separated into the expression_low_ group and the expression_high_ group as per the midvalue of the CYP1B1-AS1 expression, and subsequently, the K-M approach was leveraged to contrast the diversity of survival between these 2 groups. *p* < 0.05 had significance on statistics. We drew a curve of survival for CYP1B1-AS1 in tumors meeting the standards. We utilized Spearman's coefficient to examine the association between CYP1B1-AS1 and the relevant targeted genes CYP1B1 in pancancer. The coefficient of association > 0.4 and the *p* result < 0.001 had significance on statistics.

## 3. Results

### 3.1. The Identification of Survival-Related eRNAs in GBM

**Figure 1:**
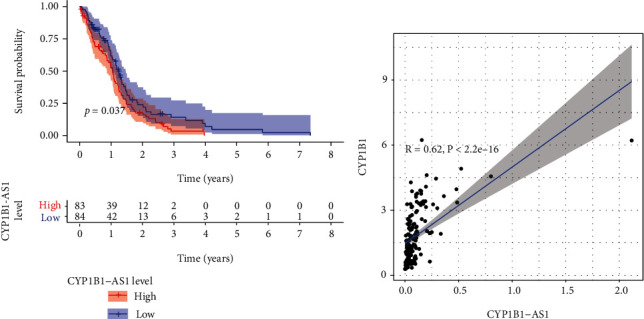
**(**a) Kaplan-Meier curve analysis of CYP1B1-AS1 for the overall survival in GBM patients from TCGA datasets. (b) There is a significant correlation between CYP1B1-AS1 expression and CYP1B1 expression in GBM patients.

To identify survival-related eRNAs in GBM, we analyzed TCGA datasets and identify 74 survival-related eRNAs by the use of Kaplan-Meier methods (Table S1). Afterwards, we utilized Spearman's correlation to select 174 eRNAs for the purpose of identifying eRNAs with a remarkable association with the targeted genes related to GBM. Merely 39 eRNAs reached the standard (Spearman's rank relational coefficient *r* > 0.40, *p* < 0.001; Table S2), and CYP1B1-AS1 displayed the greatest cor result and was hence deemed as the most correlated eRNA related to the targeted genes. K-M methods revealed that sufferers with increased expression of CYP1B1-AS1 displayed a poorer OS than those with low CYP1B1-AS1 expression (Figure 1). Moreover, a positive association existed between CYP1B1-AS1 and the targeted gene CYP1B1 (*r* = 0.62, *p* = 2.2 × 10*e* − 15; Figure 1(b)).

### 3.2. Genetic Enrichment Assay

**Figure 2:**
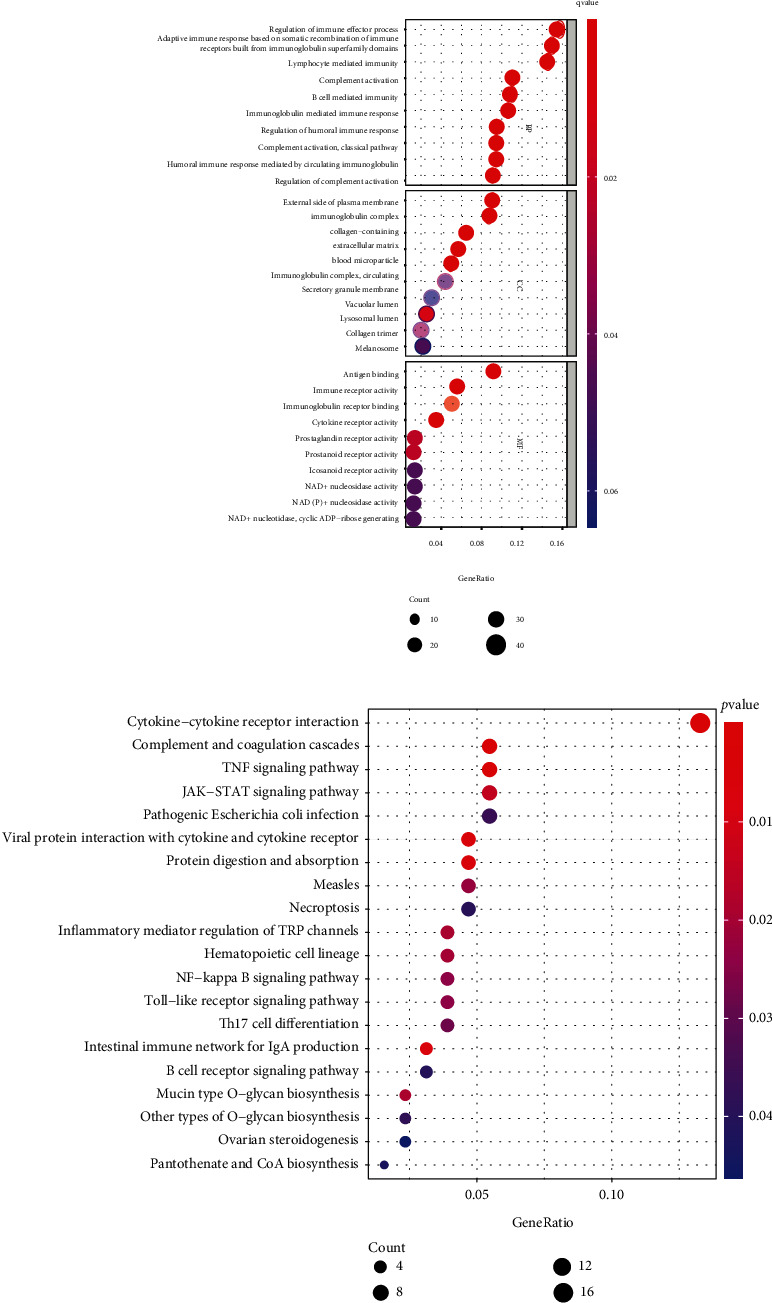
(a) GO analysis of 323 targeting genes of CYP1B1-AS1. (b) The top 20 enriched KEGG pathways.

Overall, 323 transcriptions displayed a remarkable association with CYP1B1-AS1 (*p* < 0.05), involving CYP1B1. GO enriching assay and KEGG assay of the 323 targeting genes offered the foundation for the biology research. The top 10 terms for MF, BP, and CC are presented by Figure 2(a). In BP, the terms were predominantly associated with the modulation of immunity effector processes, lymph cell-mediation immunity, complement stimulation, and B cell-mediated immunity. In CC, the terms were primarily associated with outer plasmatic membranes, immunoglobulin complexes, and collagen-involving and exocellular matrices. In MF, the terms were mainly related to antigen binding, immunoreceptor activities, immunoglobulin receptor binding, and CKR activities. Further KEGG assays revealed that the most vital pathways included CK-CKR mutual effects, complementing and coagulating cascades, TNF signal path, and JAK-STAT signaling pathway (Figure 2(b)).

### 3.3. Pancancer Validation

**Figure 3:**
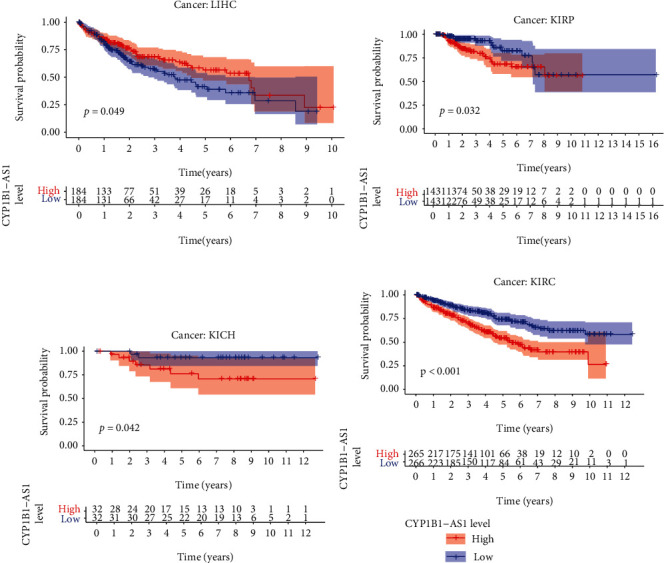
Kaplan-Meier survival curves for CYP1B1-AS1 in (a) LIHC, (b) KIRP, (c) KICH, and (d) KIRC using pancancer.

**Figure 4:**
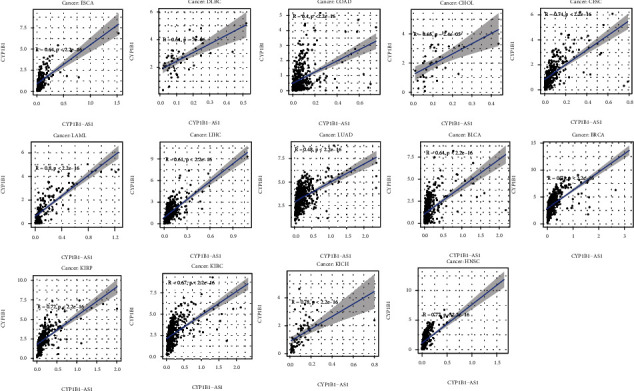
Correlations between CYP1B1-AS1 and CYP1B1 expression in ESCA, DLBC, COAD, CHOL, CESC, LAML, LIHC, LUAD, BLCA, BRCA, KIRP, KIRC, KICH, and HNSC.

**Figure 5:**
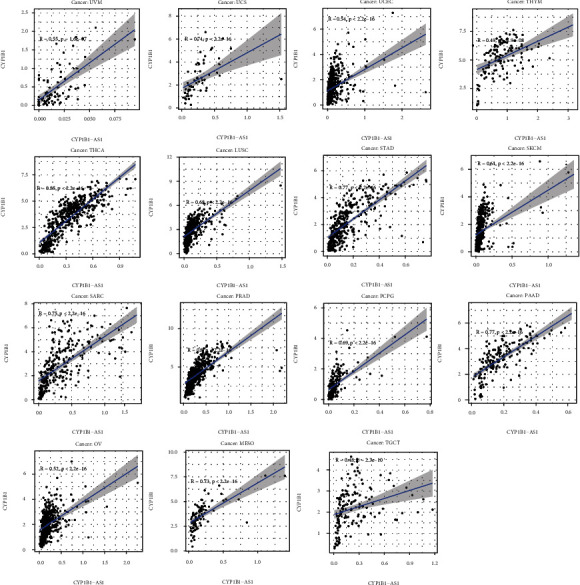
Correlations between CYP1B1-AS1 and CYP1B1 expressions in UVM, UCS, UCEC, THYM, THCA, LUSC, STAD, SKCM, SARC, PRAD, PCPG, PADD, OV, MESO, and TGCT.

For the purpose of determining the prognosis function of the screened eRNA in pancancer and the association with the targeted gene, our team completed survival and association assays. The outcomes revealed that CYP1B1-AS1 was related to survival in 4 tumors, i.e., LIHC (Figure 3(a)), KIRP (Figure 3(b)), KICH (Figure 3(c)), and KIRC (Figure 3(d)). Moreover, our team discovered that CYP1B1-AS1 and the targeted gene are related to 29 kinds of tumors (Figures 4 and 5 and Table S3).

## 4. Discussion

Tumors are still a serious threat to mankind, and tumor prevalence has exhibited a rising tendency in recent years [15]. However, the metastatic process in tumor sufferers remains elusive, although it can forecast poor prognostic results. At present, for the purpose of estimating cancer metastatic ability, determining new molecule-level biomarkers is imperative, as those biomarkers are pivotal for tumor therapies and forecast [16, 17]. eRNAs pertain to those molecule-level biomarkers, which can affect the progression and initiation of cancer; moreover, they might be easily collected for the purpose of monitoring and diagnosing cancers [18, 19].

In this study, we analyzed TCGA datasets and identified 74 survival-related eRNAs in GBM. The 74 eRNAs may be involved in the progression of GBM. Then, our attention focused on CYP1B1-AS1. In recent years, several studies have reported that eRNAs played an important role in tumor progression, including GBM. For instance, AP001056.1 was reported to be associated with long-term survivals of patients with HNSCC [20]. Enhancer RNA SLIT2 was shown to be lowly expressed in breast cancer, and its knockdown suppressed the proliferative and metastatic abilities of breast oncocytes via modulating P38 MAPK/c-Fos signal path [21]. Enhancer RNA MARC1 was found to be overexpressed in bladder cancer, which facilitated the proliferative, migratory, and invasive abilities of bladder cancer cells [22]. CYP1B1-AS1 was a newly identified eRNA which was found to be dysregulated in lung adenocarcinoma and acute myeloid leukemia [23, 24]. However, its function has not been investigated in the above tumors. In addition, the possible roles of CYP1B1-AS1 in GBM were also not investigated. In this study, we confirmed CYP1B1 as its regulatory target. The expression of CYP1B1, a component of CYP super family, exists in hepatic and extrahepatic samples, and it is responsible for the metabolic process of massive xenobiotics. In recent years, several studies have reported that CYP1B1 G119T polymorphic status might be associated with hereditary predisposition in Asian individuals, particularly when it comes to breast cancer and prostate carcinoma, suggesting the potential roles of CYP1B1 in tumor progression. However, its function in GBM remained unclear.

In recent years, many studies have used GO and KEGG assays to explore the potential function of genes in disease development [25, 26]. In this study, we identified 323 potential targeting genes of CYP1B1-AS1, which was further used for GO and KEGG assays. We found the 323 genes were positively associated with lymphocyte-mediated immunity, regulation of immune effector process, and immune receptor activity, highlighting the possible regulatory function of CYP1B1-AS1 in immune activity. In addition, KEGG pathway results showed that 323 genes were primarily enriched in TNF signal path, JAK-STAT signal path, and Toll-like receptor signal path, suggesting CYP1B1-AS1 may be involved in tumor progression of GBM [27–29]. Moreover, we performed pancancer assays, finding that CYP1B1-AS1 was related to survival in 4 kinds of cancers (LIHC, KIRP, KICH, and KIRC). In addition, the expression of CYP1B1-AS1 was related to the expression of the targeted gene, CYP1B1, in 29 cancer types. Holistically, the outcomes in the present research revealed that CYP1B1-AS1 can be utilized as an independent predicting factor of GBM.

## 5. Conclusion

We identified 74 survival-related eRNAs in GBM. CYP1B1-AS1 is pivotal for the BP of cancer development through various pathways, and its overexpression might be used as a prognostic biomarker for GBM patients.

## Data Availability

The data used to support the findings of this study are available from the corresponding author upon request.

## Abbreviations

eRNAs: Enhancer RNAs
GBM: Glioblastoma
TCGA: The Cancer Genome Atlas
FDR: False discovery rate
MF: Molecular functions
CC: Cellular components
BP: Biological processes
CYP1B1: Cytochrome P450 1B1
CNS: Central nervous system
K-M: Kaplan-Meier
OS: Overall survival
HNSCC: Head and neck squamous cell carcinoma
CKR: Cytokine receptor
CK: Cytokine
KEGG: Kyoto Encyclopedia of Genes and Genomes.

## Supplementary Materials

Supplementary 1 Table S1: list of 75 survival-related eRNAs in GBM.

Supplementary 2 Table S2: list of the 40 eRNAs with a significant correlation with their target gene in GBM.

Supplementary 3 Table S3: list of the 30 types of tumors associated with CYP1B1-AS1 and CYP1B1.
