# Supplementary material for: CYP1B1-AS1 Is a Novel Biomarker in Glioblastoma by Comprehensive Analysis
Source: Dis Markers. 2021 Dec 29;2021:8565943. doi: 10.1155/2021/8565943 (PMC8733712; doi:10.1155/2021/8565943)
Supplement: Supplementary 1 — Table S1: list of 75 survival-related eRNAs in GBM. [file 8565943.f1.pdf]

Table S1. List of 75 survival-related eRNAs in GBM.

gene	KM	gene	KM
AL356215.1	0.014281	LINC02026	0.034819
LINC01285	0.016247	AL390198.1	0.018933
LINC01615	0.004159	AC103702.2	0.042944
LINC00354	0.047878	AC124852.1	0.0371
LINC00886	0.004709	IGHA2	0.01166
FAM120AOS	0.044716	MIR217HG	0.012982
LEF1-AS1	0.000246	AL021937.1	0.006565
ZBED3-AS1	0.036844	LBX1-AS1	0.025797
AC015909.1	0.007557	AC083864.2	0.044705
LINC02036	0.015917	LINC01088	0.023801
LINC01111	0.018822	AC113346.1	0.042853
SLC44A3-AS1	0.035431	AP003555.1	0.01921
ZNF337-AS1	0.006093	ALDH3B1	0.005684
AC069281.1	0.025099	AP001471.1	0.038946
GCC2-AS1	0.001963	AC078785.1	0.041339
AP003469.2	0.017891	AC007405.2	0.043425
ZNRF2P2	0.039869	LINC01529	0.034932
BX284668.2	0.043445	AP000424.1	0.032463
HOTAIR	0.015604	LRRC8C-DT	0.044126
CRNDE	0.003139	AP002761.1	0.035427
AC092164.1	0.008008	EDNRB-AS1	0.049895
OSMR-AS1	0.017708	LINC01248	0.046692
LINC01574	0.003659	LY6E-DT	0.038297
MYOSLID	0.007847	AC107223.1	0.004862
AC004923.4	0.006884	AL158151.1	0.003797
PROX1-AS1	0.00902	AC018866.2	0.000764
CHST12	0.018207	ZMIZ1-AS1	1.28E-05
AC073316.2	0.041095	AC003092.1	0.016754
AF015262.1	0.023739	LINC00665	0.016429
LINC02324	0.016507	AL355607.1	0.036478
CYP1B1-AS1	0.037351	LINC02154	0.042814
AC104596.1	0.045214	MATN1-AS1	0.008756
LINC02773	0.004334	HOXC-AS3	0.004981
APELA	0.018438	CYP4A22-AS1	0.046964
SPRY4-AS1	0.006354	LINC00398	0.039217

AC125613.1	0.047381	AC093772.1	0.009892
LINC02577	0.028153	AC013287.1	0.024223
