# Supplementary material for: CYP1B1-AS1 Is a Novel Biomarker in Glioblastoma by Comprehensive Analysis
Source: Dis Markers. 2021 Dec 29;2021:8565943. doi: 10.1155/2021/8565943 (PMC8733712; doi:10.1155/2021/8565943)
Supplement: Supplementary 2 — Table S2: list of the 40 eRNAs with a significant correlation with their target gene in GBM. [file 8565943.f2.pdf]

Table S2**.** List of the 40 eRNAs with a significant correlation with their target gene in GBM.

eRNA	KM	Target	cor	corPval
SLC44A3-AS1	0.035431	SLC44A3	0.703909	0
SLC44A3-AS1	0.035431	CNN3	0.554292	0
SLC44A3-AS1	0.035431	F3	0.436655	4.75E-09
PROX1-AS1	0.00902	PROX1	0.525114	0
MATN1-AS1	0.008756	MATN1	0.450114	1.38E-09
LINC02773	0.004334	BATF3	0.537959	5.49E-14
LINC01248	0.046692	SOX11	0.508528	1.96E-12
CYP1B1-AS1	0.037351	CYP1B1	0.615817	0
LINC01088	0.023801	PAQR3	0.440499	3.37E-09
LINC01088	0.023801	NAA11	0.7209	3.14E-28
LEF1-AS1	0.000246	LEF1	0.602401	0
ZBED3-AS1	0.036844	PDE8B	0.420478	1.92E-08
ZBED3-AS1	0.036844	ZBED3	0.494012	5.58E-12
SPRY4-AS1	0.006354	SPRY4	0.500407	0
AC113346.1	0.042853	MSX2	0.415599	2.12E-08
LINC01574	0.003659	SNCB	0.508427	1.98E-12
LINC01574	0.003659	UNC5A	0.4694	1.38E-10
CHST12	0.018207	IQCE	0.537409	0
AC083864.2	0.044705	EEPD1	0.598241	1.10E-17
AC003092.1	0.016754	TFPI2	0.597939	1.15E-17
AP003555.1	0.01921	ANO1	0.539298	4.62E-14
AL356215.1	0.014281	CD44	0.664652	8.94E-23
HOTAIR	0.015604	HOXC10	0.832982	1.59E-44
HOTAIR	0.015604	HOXC11	0.911022	9.26E-66
HOTAIR	0.015604	HOXC13	0.824268	7.36E-43
HOTAIR	0.015604	HOXC6	0.671807	2.11E-23
HOXC-AS3	0.004981	HOTAIR	0.795494	5.96E-38
HOXC-AS3	0.004981	HOXC11	0.820983	2.96E-42
HOXC-AS3	0.004981	HOXC13	0.728099	5.02E-29
HOXC-AS3	0.004981	HOXC6	0.739979	2.14E-30
CRNDE	0.003139	IRX5	0.746936	0
LINC00665	0.016429	ZFP14	0.623337	0
LINC00665	0.016429	ZFP82	0.459271	5.69E-10
LINC00665	0.016429	ZNF146	0.495282	3.88E-12
LINC00665	0.016429	ZNF260	0.739541	0

ZNF337-AS1	0.006093	NINL	0.431535	7.46E-09
AP001471.1	0.038946	COL6A1	0.603759	4.60E-18
AP001471.1	0.038946	COL6A2	0.671878	2.08E-23
AL021937.1	0.006565	RFPL3	0.452123	7.65E-10
