# Supplementary material for: CYP1B1-AS1 Is a Novel Biomarker in Glioblastoma by Comprehensive Analysis
Source: Dis Markers. 2021 Dec 29;2021:8565943. doi: 10.1155/2021/8565943 (PMC8733712; doi:10.1155/2021/8565943)
Supplement: Supplementary 3 — Table S3: list of the 30 types of tumors associated with CYP1B1-AS1 and CYP1B1. [file 8565943.f3.pdf]

Table S3. List of the 30 types of tumors associated with CYP1B1-AS1 and CYP1B1.

eRNA	Target	cancerType	cor	corPval
CYP1B1-AS1	CYP1B1	BLCA	0.636178	5.26E-48
CYP1B1-AS1	CYP1B1	BRCA	0.713951	0
CYP1B1-AS1	CYP1B1	CESC	0.741945	0
CYP1B1-AS1	CYP1B1	CHOL	0.654054	2.41E-05
CYP1B1-AS1	CYP1B1	COAD	0.40391	6.56E-20
CYP1B1-AS1	CYP1B1	DLBC	0.608988	7.00E-06
CYP1B1-AS1	CYP1B1	ESCA	0.636957	0
CYP1B1-AS1	CYP1B1	GBM	0.615817	0
CYP1B1-AS1	CYP1B1	HNSC	0.768031	7.87E-99
CYP1B1-AS1	CYP1B1	KICH	0.778059	0
CYP1B1-AS1	CYP1B1	KIRC	0.669122	0
CYP1B1-AS1	CYP1B1	KIRP	0.715333	0
CYP1B1-AS1	CYP1B1	LAML	0.804346	0
CYP1B1-AS1	CYP1B1	LIHC	0.608553	2.91E-39
CYP1B1-AS1	CYP1B1	LUAD	0.481712	0
CYP1B1-AS1	CYP1B1	LUSC	0.67923	4.79E-69
CYP1B1-AS1	CYP1B1	MESO	0.732006	0
CYP1B1-AS1	CYP1B1	OV	0.518474	0
CYP1B1-AS1	CYP1B1	PAAD	0.766236	0
CYP1B1-AS1	CYP1B1	PCPG	0.686669	7.37E-27
CYP1B1-AS1	CYP1B1	PRAD	0.796631	0
CYP1B1-AS1	CYP1B1	SARC	0.746608	0
CYP1B1-AS1	CYP1B1	SKCM	0.644331	1.34E-56
CYP1B1-AS1	CYP1B1	STAD	0.766112	0
CYP1B1-AS1	CYP1B1	TGCT	0.483479	2.31E-10
CYP1B1-AS1	CYP1B1	THCA	0.851273	0
CYP1B1-AS1	CYP1B1	THYM	0.493106	1.90E-08
CYP1B1-AS1	CYP1B1	UCEC	0.539296	1.13E-42
CYP1B1-AS1	CYP1B1	UCS	0.737867	0

CYP1B1-AS1	CYP1B1	UVM	0.546204	1.60E-07
